# A Reply to Some Views on the Putrefactive Theory of Decay

**Published:** 1884-01

**Authors:** W. D. Miller

**Affiliations:** Berlin


### A REPLY TO SOME VIEWS ON THE PUTREFACTIVE THEORY OF DECAY.

BY W. D. MILLER, BERLIN.

In the November number of the *New England Journal of Dentistry* will be found an article by Dr. C. T. Stockwell, Springfield, Mass., on "Micro-organisms the Essential Factor in Dental Caries."

There are two sources of error to which every experimenter is exposed; he may err in the work itself, and he may err in the interpretation of the results of his work. Such errors are of course pardonable, unless resulting from pure carelessness, or pre-formed opinion. One occupying the position of Dr. Stockwell, who claims not to present original investigations, but "logical deductions"

from the investigations of others, is liable even in a greater degree to the same errors, because not having performed the work he cannot possibly enter into the real significance of it as well as the experimenter himself. He is, moreover, liable to additional errors in misunderstanding or improperly interpreting the work of the author, and in unconsciously picking out from that work those portions which best suit his logical deductions. All of these errors Dr. Stockwell has fallen into. Especially should his conclusions be accepted with reserve when they differ from those of the investigator, or when they are based upon no experiments at all.

Dr. Stockwell concludes the whole matter at the beginning by a very convenient definition of the subject: "Dental caries does not begin until the putrefactive process of an organic portion of the tooth has been set up." If we say that the death of an animal does not occur till the worms begin to consume its body, then, if you please, maggots caused the death of Polonius; but according to common acceptation a thrust from Hamlet's rapier ended the life of that statesman. Putrefaction is not the first, but the last stage of dental caries. Where Dr. Stockwell got his authority for the contrary he does not state. I have pieces of enamel and dentine which, since May 16th, 1882, have been in contact with putrefying substances at blood temperature and proper conditions of moisture. (Such a piece is now in possession of Dr. Ross, of Chicopee, Mass.) They show no trace of caries, and when, then, any one says, without the slightest semblance of proof for the assertion, that putrefaction of itself produces caries, I cannot respect his statement.

I have given, in the INDEPENDENT PRACTITIONER for December, '83, a method by which I have (and every one can) produced caries, which to the naked eye as well as under the microscope is not to be distinguished from natural decay. Dr. Barrett has such preparations in his possession.

The characteristic phenomena of caries begin to show themselves in ten weeks. Now I challenge Dr. Stockwell, and all those of his or any other belief, to discover and publish a process by means of which, without the aid of acids, (by putrefaction alone), one may accomplish what I have done. Then, and not until then, can they demand that their views be accorded a serious hearing.

I have perhaps done my part to place bacteria on a respectable footing in dentistry. Two and a half years ago, at the meeting of American dentists in Wiesbaden, I pointed out the part which putrefaction plays in caries; but putrefaction does not by any means occupy the first place in the process.

Dr. Stockwell's distinction between fermentation and putrefaction contains some grave errors; he says: "Remember that the product of fermentation is always an acid, while the product of putrefaction is always alkaline, and that both processes are impossible where micro-organisms are eliminated."

Dr. Stockwell has evidently overlooked the whole science of Enzymology, which treats only of non-organized (chemical) ferments; we should soon run ashore if we were to ascribe, without distinction, all cases of fermentation to fungi. He has also forgotten that fungi often contain other ferments than such as produce acid. Many contain a ferment similar to diastase (*clostrydium butyricum*); some a ferment like invertine (*leuconostoc mesenterioides*); one at least contains a ferment which dissolves coagulated albumen and converts it into peptone, (*bacterium subtile*); some even contain a ferment which produces ammonia (*micrococcus ureae*, Cohn—*Ferment der Ammoniak*, Gâhrung), etc., etc. I have claimed for many months that the acid of tooth-caries is produced in the mouth by fermentation, and was for a long time uncertain whether the ferment was organized or chemical; it required a vast amount of labor to show that it was the former.

That part of Dr. Stockwell's statement which is sufficiently accurate, viz: "the product of putrefaction is always alkaline," contains in itself a complete refutation of the one point which he would like to establish. The product of putrefaction being always alkaline, then if caries is simply putrefaction, decaying dentine should always have an alkaline reaction, while as a matter of fact its reaction is constantly acid. Thus unwittingly does the writer annihilate his own theories. Farther on Dr. Stockwell says: "Nothing has been found beyond the organisms." If he had made and examined a few preparations of carious dentine, he could not have entertained this idea for a moment. Beyond the organisms has been found a zone of softened, partially decalcified dentine, *which may be readily cut with a razor.* You may put a thousand million little "devils" at the end of each dentinal fibril, if you like, to "absorb the protoplasm from the fibrils," and I am afraid the dentine would remain as hard as before. Dr. Stockwell still labors under the false assumption that no decalcification takes place in decay, than which a greater error never hampered a theory of dental caries. The decalcification of the outer layers is almost absolute, and gradually decreases as we approach the normal dentine. Exactly the same is the case with dentine slowly decalcified in saliva and bread. Dr. Stockwell next asks: "What are they after?" *i. e.* what are the organisms after in carious dentine? It hardly seems necessary to answer this question. He knows as well as I do that the decalcified dentine becomes soaked in the fluids of the mouth; the organisms may live at the expense of these fluids, and when the decalcification has progressed far enough they may also live at the expense of the softened dentine itself.

On page 352, Dr. Stockwell tells us what a micrococcus may do to sound enamel. Unfortunately he does not give the source of his authority for these statements. I would ask him if *he* ever saw a micrococcus, or any other species of micro-organism making its way into enamel which was not already in a state of partial disintegration, and if he did not, who did? I have scores of preparations of carious enamel, and have already stated that in caries of the enamel micro-organisms are agents of no consequence whatever, except in as far as they by fermentation may lead to the production of acid. I should like to know where the logical deduction comes in.

At the bottom of page 351, Dr. Stockwell confuses one of my statements, and comes at last to the conclusion that "if the invading agent was a destructive acid the tortuosity and angularity would never appear at all." If the writer will refer to my article he will see that this is exactly the case, as I stated it, "so that one may draw a curved line through the preparation and say: on one side is normal, on the other softened dentine." The fungi advance irregularly, the decalcification with considerable regularity. It is a serious error, when one does not investigate himself, but on the contrary perverts the work of others.

At the bottom of page 352, Dr. Stockwell again gives expression to his convictions that everything which precedes putrefaction is not to be regarded as part of the carious process; *i. e.* the enamel of a tooth may be destroyed, the dentine decalcified and softened, and its organic portion permeated with oral secretions and every way laid bare to decomposing agents, to which it was previously invulnerable; all this, according to Dr. Stockwell, would not be called the beginning of caries. A man receives a severe wound in the abdomen and dies some hours later of peritonitis; we say that his death was caused by a wound in the abdomen. Every tooth must be very severely wounded by acids before putrefaction sets in; the wound is the cause, putrefaction only one of the later stages in the disease, and in this case can only occur in consequence of, or subsequent to the wound.

At the middle of page 353, the doctor gets a glimpse of the truth, but loses it immediately afterward on the bacterium termo. I would like to ask Dr. Stockwell if he is sure that bacterium termo is found in considerable numbers in carious dentine. Is he sure that it is found at all at the head of the invasion? Has he ever obtained it in cultures of carious dentine, and if so, has he found that it has at all the power of softening a piece of dentine so that it may be cut with a razor? Is he aware that later experiments have rendered it doubtful whether bacterium termo is at all to be considered as a true putrefactive bacterium; that for decomposition, associated with offensive odor, the presence of other fungi is necessary? *

Can he give a single reason for considering bacterium termo as *the* one factor in dental caries? If he cannot, why does he invest it with this power?

I have been able to put together the following chain of reasoning from the dental journals: Bacterium termo is found in putre-

* Cohn's Beitr. 1 Bd. 3 Heft S. 214.—Fluegge Handb. d. Hygiene, S. 112.

fying substances (blood, etc.); something which looks like bacterium termo is found in the human mouth,* therefore bacterium termo is the cause of dental caries. If Dr. Stockwell can give other reasons I shall be glad to hear them. Bacterium termo mixed up with other fungi may, no doubt, occur in the human mouth; what of that! there is material enough there for it to feed upon. *Prove* that it has the power to attack sound teeth. It is the complete lack of even an attempt to establish one's views by exact experiment, that characterizes nearly all articles on the putrefactive theory of caries, which have appeared in the dental journals. We would recommend to those who maintain that a purely putrefactive bacterium with its alkaline products is *the* cause of dental caries: 1st. To establish by experiment that such a bacterium exists in considerable numbers in the deeper parts of carious dentine.

2d. To determine the species of said bacterium.

3d. To isolate it by pure culture and study its morphology, physiology, developement, etc., etc.

4th. To *prove* that in pure culture it can cause the decay of enamel and dentine.

If they can do this then, *a la bonne heure*, we shall be ready to give them due praise. If they attempt it without succeeding, the thanks of the profession will still be due them for their work, but if they do not even make the attempt, and go on with ungrounded assertions all the same, then we can pay no respect to such statements, nor can the scientific world regard them. The fact that antiseptics have been used for years with great success in dentistry, does not begin to prove that caries is simply a putrefactive process. Antiseptics, also avert, or completely stop the production of acid by fermentation.

